# Impact of power ultrasound on the quality of leafy green produce through a multifrequency, multimode, modulated system

**DOI:** 10.1016/j.ultsonch.2024.107221

**Published:** 2025-01-02

**Authors:** Bin Zhou, J. Atilio de Frias, Yaguang Luo, Jorge M. Fonseca, Hao Feng

**Affiliations:** a Food Quality Lab, Beltsville Agricultural Research Center, United States Department of Agriculture, Beltsville, MD 20705, USA; b Enviromental Microbial and Food Safety Lab, Beltsville Agricultural Research Center, United States Department of Agriculture, Beltsville, MD 20705, USA; c Department of Family and Consumer Sciences, North Carolina Agricultural and Technical State University, Greensboro, NC 27411, USA

**Keywords:** Ultrasound, Fresh produce, Leafy greens, Quality

## Abstract

Ultrasound technology has been increasingly explored as an eco-friendly method to improve the microbial safety of leafy greens. However, its effect on produce quality is critical, and considerable knowledge gaps remain in this area. The present study examined the response of leafy greens to ultrasound treatment as shown by tissue damage and sensory quality, using a novel multifrequency, multimode, modulated (MMM) system to address the issue of nonuniform ultrasound field distribution. Iceberg lettuce, romaine lettuce, spinach, loose leaf lettuce and Lollo Rosso were subjected to different ultrasonication durations (1–16 min) in a MMM tubular treatment unit at 34 kHz and subsequently stored at 1 °C for three weeks. Sensory evaluations by a trained panel and electro-conductivity rate measurements were conducted to assess produce quality over time. Ultrasound treatment at an acoustic power density below 80 W/L had no significant effect (P > 0.05) on the overall sensory quality of leafy greens during 14 days of storage. Even though the electro-conductivity rate, an indicator of tissue damage, increased in ultrasound-treated samples compared to control, it did not result in perceptible changes in sensory attributes.

## 1 Introduction

The fresh-cut produce industry has experienced sustained growth due to high consumer demand for convenient and healthy food options [1]. However, the sector faces significant challenges related to food safety, as pathogen-contaminated leafy greens have been the source of numerous foodborne illness outbreaks. Commercial scale washing methods are generally safe but have proven insufficient in significantly reducing food safety risks.

Ultrasound treatment can potentially enhance produce safety through microbial inactivation due to acoustic cavitation-driven activities [2], [3], [4], [5]. However, the collapse of cavitation bubbles generates localized extreme temperatures and pressures, which may damage plant tissues [6], [7], [8]. In addition, acoustic microstreaming caused by oscillating bubbles creates shear forces that contribute to cell damage. The acoustic power density (APD) and frequency of ultrasound are factors influencing these effects, with higher levels correlating to increased damage [9], [10], [11]. A critical consideration for the application of ultrasound regarding both microbial inactivation and produce quality is the nonuniform distribution of ultrasound inside a treatment unit/chamber. The nonuniform distribution of the ultrasound field, and consequently the uneven cavitation, leads to variations in microbial inactivation across different locations inside a produce washing tank. As a result, samples that have received adequate ultrasonication and show low microbial counts can be easily cross contaminated by neighboring samples that have not undergone sufficient ultrasound treatment due to the uneven sound field distribution [12]. Similarly, a produce sample at the 'hot spot' of an ultrasound field may receive excessive ultrasound exposure, resulting in greater tissue damage. It is known that ultrasound field distribution in both conventional probed-type and bath-type ultrasound devices is non-uniform. Therefore, in studies aimed at understanding how ultrasound impacts produce quality, measures must be taken to address the non-uniform ultrasound distribution.

In this study, a uniform ultrasound field is achieved by the multifrequency, multimode, modulated (MMM) ultrasound generation technique. An MMM ultrasonic unit consists of a sweeping-frequency, adaptively modulated waveform power supply; an acoustic waveguide, which connects the ultrasonic transducer to an acoustic load; an acoustic load (mechanical resonating tubular cavity); and sensors of acoustic activity fixed on the tubular cavity, creating regulation feedback between the acoustic load and the ultrasonic power supply. The MMM ultrasonic converter, driven by the power supply, produces a strong pulse-repetitive multifrequency train of mechanical pulses. The acoustic load, driven by the incoming frequency and amplitude-modulated pulse train, begins to produce its own vibrations and transient responses, oscillating in one or more of its natural vibration modes or harmonics, thereby generating multifrequency, multimode, modulated ultrasound with uniform sound distribution [13].

To effectively utilize ultrasound for produce decontamination, a careful balance must be achieved between microbial inactivation and product quality preservation. Optimizing treatment parameters, such as acoustic power density (APD), exposure time, and frequency, is crucial to mitigate potential damage while ensuring adequate pathogen reduction. Furthermore, understanding the intricacies and characteristics of different produce types is key for developing tailored ultrasound applications. Recent studies have investigated the effects of ultrasonication on lettuce, spinach, and berries [14], [15], providing valuable insights on improving produce safety without compromising quality.

This study explored the impact of ultrasound on the quality of five leafy green types, e.g., iceberg lettuce, romaine lettuce, loose-leafy lettuce, baby spinach, and Lollo Rosso lettuce during storage, employing advanced MMM ultrasound technology to achieve uniform distribution in a tubular treatment unit. We determined the acoustic power density (APD) thresholds of leafy greens that did not affect product quality after two weeks of storage. The quality assessment included the evaluation of electrolyte leakage, color, texture, and sensory attributes of the produce samples.

## 2 Materials and methods

### 2.1 Multifrequency, multimode, modulated (MMM) ultrasound treatment of produce and quality assessments

**Fig. 1:**
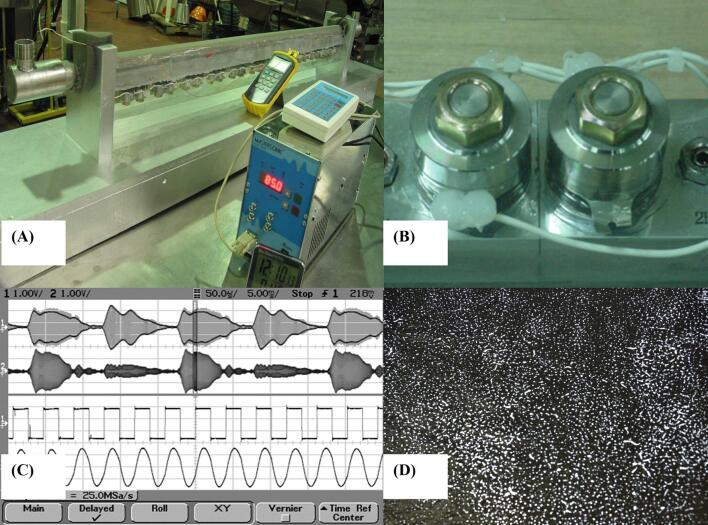
Multifrequency, Multimode, Modulated Sonic and Ultrasonic system. (A) photo of the MMM tubular treatment unit; (B) ultrasonic transducers; (C) frequency and waveform diagrams; and (D) aluminum foil treated in MMM system.

To test the effect of ultrasound treatment time on produce quality, a multifrequency, multimode, modulated (MMM) system (M P Interconsulting, Switzerland) was used at a central frequency of 34 kHz and an acoustic power density of 81 W/L (Fig. 1A). Baby spinach, loose-leafy lettuce, Lollo Rosso, and romaine lettuce were transported overnight from a supplier in California to our research laboratories at the U.S Department of Agriculture-Agricultural Research Service in Beltsville, MD. The MMM reactor had an inner diameter of 38.1 mm, and a custom sample holder was designed to secure the cut produce samples during treatment. For romaine lettuce and loose-leafy lettuce, two outer layers were removed, and inner layers were cut into 25.4 × 25.4 mm pieces. Baby spinach and Lollo Rosso leaves were treated whole. One-hundred-gram samples were placed in the sample holder, which was then inserted into the tubular treatment unit filled with pre-cooled tap water at 5 °C. After treatment under different treatment times (1, 2, 4, 8, and 16 min), the water was drained, and the holder was removed. Samples were tested for electro-conductivity rate (ECR).

The ECR data analysis and curve-fitting were conducted following a modified 4-parameter Sigmoidal Gompertz model (Eq. (1) and using SigmaPlot 12.3 (Systat Software Inc. San Jose, CA) to plot the curves for each type of produce:

(1) $$Y\left(E\right)=y_{0}+a\times exp\left\{-exp\left[\frac{-(E-E_{0})}{b}\right]\right\}$$

In Eq. (1), *Y(E)* represents ECR (%) at input energy density *E*; *E_0_* represent the input energy density at the inflection point; y *_0_* represents the asymptotic ECR as E approaches zero; *a* represents the asymptotic ECR as *E* increases, and 1/*b* represent the relative increase rate at *E_0_*.

To test the effect of ultrasound treatment on produce quality during post-harvest storage, produce samples were treated with the MMM system under different treatment times (0, 1, and 2 min), and packaged with oxygen transmission rate (OTR) film of 8.0 pmol O_2_/s·m^2^·Pa and stored for three weeks at 1 °C. Samples were tested for color, texture, ECR, and sensory evaluation on days 0, 7, 14 and 21.

### 2.2 Tissue and membrane integrity

Tissue and membrane integrity were assessed by measuring electrolyte leakage [16]. Five-gram samples were removed from each treatment and submerged in 50 mL of deionized water for 60 min at 23 °C. Samples were agitated using a shaker at 100 rpm. Electrical conductivity of the bathing solution was measured at 1 min (C1) and 60 min (C60) of incubation using a conductivity meter (Model Accumet Basic AB30, Thermo Fisher Scientific, Waltham, MA). Samples were then autoclaved (121 °C) for 25 min, and total conductivity (CT) of the bathing solution was measured after cooling. Electrolyte leakage (E) was calculated as follows:

(2) $$E=\frac{\left(C60-C1\right)}{\mathrm{CT}}\times 100$$

### 2.3 Color assessment

To assess changes in produce color, a Minolta CR-300 Chroma Meter (Minolta Corporation, Ramsey, NJ, USA) was used based on the CIELAB color system. The color meter was calibrated using standard white and black plates. Color readings were taken from different parts of the produce, such as lettuce ribs and leaves. Three readings were made on each sample from each package, and the mean values for L* (lightness), a* (redness), and b* (yellowness) were reported. Hue angle and chroma (saturation) were calculated as follows [17]:

(3) $$\mathrm{Hueangle}=\tan^{-1}\left(\frac{b*}{a*}\right)and\;Chroma=\sqrt{{a*}^{2}+{b*}^{2}}$$

### 2.4 Texture analysis

Firmness was measured using a Kramer Shear Press with five blades (TA-91) attached to a TA-XT2 Texture Analyzer (Texture Technologies Corporation, NY). Twenty-gram samples were cut into 25.4 × 25.4 mm pieces and placed in a sample holder. The five-blade plunger was forced through the sample at a downward speed of 1.0 mm/s from a height of 65 mm. The maximum force (N) and work (J) until shear (cutting) were recorded using the Texture Expert software, version 2.55 (Texture Technologies Corp., NY). Three measurements were performed for each sample at room temperature (21 °C).

### 2.5 Sensory evaluation

The sensory characteristics of produce, including overall quality, color, sogginess, and off-odor, were evaluated on days 0, 7, 14, and 21 by a panel of six trained individuals according to a modified method by Azodanlou et al. [18]. Each week, six samples (control and ultrasound-treated) were presented to each panelist. A hedonic scale (1–5) was used for overall quality assessment, with 1 being the most disliked and 5 the most liked). Color was rated on a scale of 1–5, with 1 indicating no green or severe browning and 5 indicating severe browning. Sogginess was rated from 1 (crisp) to 5 (soggy-watery), and off-odor was rated from 1 (none) to 5 (severe). The minimum scores for commercial acceptance were set at 3 for color and overall quality, and 2 for sogginess and odor. Samples were identified by three-digit codes and randomly placed on white paper plates for visual inspection only.

### 2.6 Experimental design and statistical analysis

Experiments were performed under a completely randomized two-way ANOVA design (SigmaPlot 12.3, Systat Software Inc. San Jose, CA). Each treatment was run in triplicates, with treatment times, storage times and type of produce as main factors, and electro-conductivity rates, visual quality scores, and other sensory data as dependent variables. Treatment means were compared based on the differences of least-square means and tested for significance using Sidak adjusted p-values to maintain experiment-wise error ≤ 0.05.

## 3 Results and discussion

### 3.1 Effect of treatment time on the electro-conductivity rate (ECR) of selected vegetables

The MMM system was developed to produce a uniform spatial field in acoustic power density (APD) to allow the entire available vibrating domain acoustically active while eliminating the creation of standing waves [13] (Fig. 1). In the MMM unit, two types of oscillations can be generated from the ultrasonic power supply; 1) a variable-frequency sweeping oscillation around a central operating frequency; and 2) an amplitude-modulated output signal. The frequency of amplitude modulation follows subharmonic low frequency vibrating modes of the mechanical system. The MMM technology can utilize the coupled vibrating modes in a mechanical system by applying advanced digital signal processing to create driving wave forms that synchronously excite many vibrating modes (harmonics and subharmonics) in an acoustic load. Therefore, the applicability of the MMM system to evaluate the effect of ultrasound on the quality of leafy green produce is of interest. The uniform ultrasound distribution in the MMM unit was validated with the traditional aluminum foil method. Pitting degenerated by cavitation activities can be visually observed on the surface of the aluminum foil placed inside the tubular MMM unit. Fig. 1D shows the pitting pattern to be uniform across the entire surface of the foil, which indicated uniform cavitation activity.

**Fig. 2:**
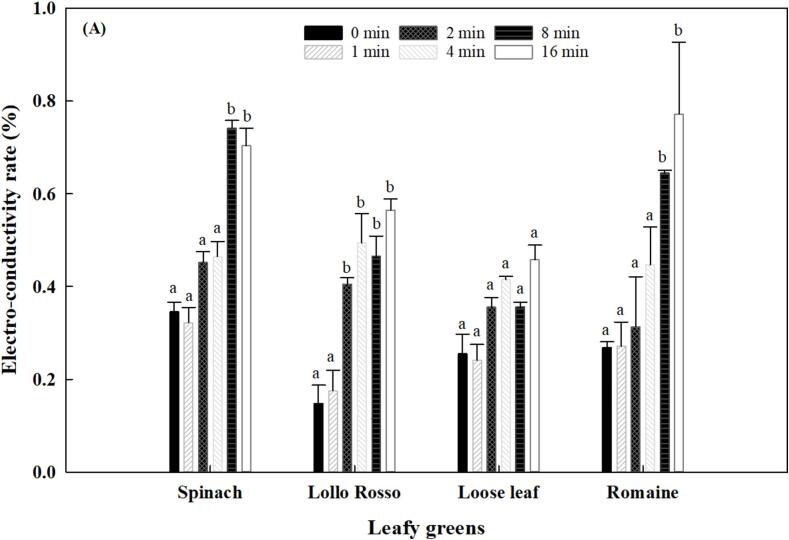
Effects of ultrasound treatment time on the electro-conductivity rate of spinach, loose leaf lettuce, Lollo Rosso, and romaine lettuce at 34 kHz and the acoustic power density of 81 W/L. Lowercase letters represent comparisons among different treatment time within the same type of selected leafy greens (P < 0.05).

Electrolyte leakage (EL) serves as one of key indicators of membrane integrity in green leafy vegetables and is closely associated with their quality, particularly during senescence and under environmental stresses [19]. This study aimed to evaluate the ECR of several selected leafy greens, including spinach, Lollo Rosso, chopped loose leaf lettuce, and chopped romaine lettuce, after ultrasound treatment. In Fig. 2, ECR for all treated vegetables increased with treatment time except for loose leaf lettuce. Ultrasonication did not result in significant changes in ECR (P > 0.05) until up to 8 min for spinach and romaine lettuce, and up to 2 min for Lollo Rosso, which indicates that a threshold for energy density input by ultrasound 648 J/L and 162 J/L, respectively. Results show two phases of ECR changes for baby spinach, loose leaf lettuce, and Lollo Rosso lettuce. In Phase I, ECR increased rapidly while in phase II, ECR changed slowly. This two-phase change of ECR may be explained by the limited penetration capacity of power ultrasound, as only plant cells and tissues that are impacted by ultrasound can be injured during ultrasonication [20]. Therefore, the efflux disruption of cell and tissue on produce surface and subsurface, results in the rapid increase of ECR in the surrounding solution in Phase I. Afterwards, the cells and tissues located at relatively deep layers receive less ultrasound exposure, hence, the permeability and integration of cells change slowly, resulting in the slow electrolyte leakage and low ECR values in Phase II. Previous studies found the penetration depth of ultrasound is limited to biological tissue, and that penetration is dependent on frequencies [20]. In our work, romaine lettuce was an exception compared to other three types of leafy greens tested [20], and ECR of fresh-cut lettuce slices increased linearly over ultrasound treatment time. This may be a result of the multiple-pores structure of fresh-cut lettuce, so ultrasound cavitation may easily take place in the inner cells. Change in the ECR of Lollo Rosso lettuce was the highest, indicating that Lollo Rosso was the most sensitive to ultrasound treatment. Even though the change of ECR was significant over treatment time (P < 0.05), no significant difference in the overall quality score was observed for all four tested vegetables after treatment (P > 0.05) (Fig. 2).

**Fig. 3:**
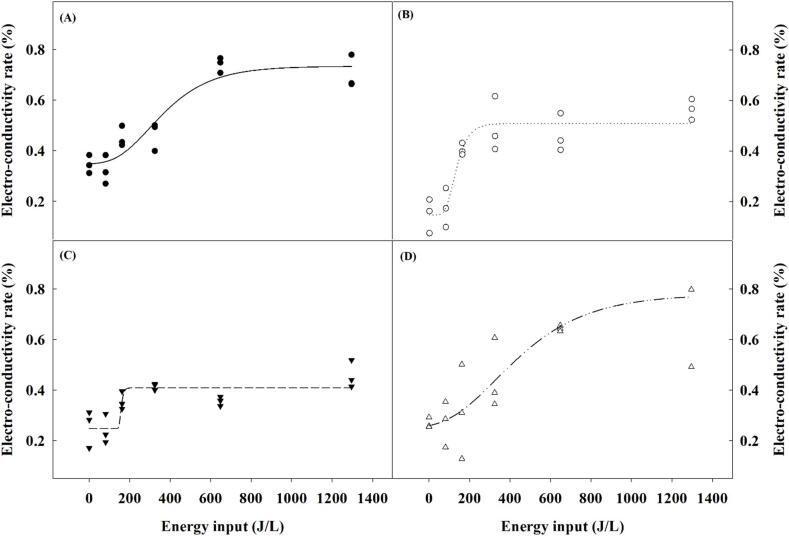
Experimental data fit with a modified Gompertz model of the electro-conductivity rate of produce versus energy input at 34 kHz and 81 W/L. (A) spinach, (B) Lollo Rosso, (C) loose leaf lettuce, and (D) romaine lettuce.

The experimental data was modeled using a 4-parameter sigmoidal Gompertz model, which effectively captured the dynamic changes in ECR across different energy inputs (Fig. 3). The ECR curves depict the characteristic two-phase response: an initial exponential increase followed by a stationary phase. This pattern is indicative of the energy absorption behavior of the greens during ultrasound treatment, where a rapid increase in ECR is observed until the material reaches a saturation point. Table 1 presents the estimated parameters for each type of produce. For spinach, parameter *a*, which represents the asymptotic increase in ECR, was estimated at 0.39 with a high level of significance (p < 0.0001). The inflection point *E_0_* was found at 298.34 J/L with a standard error of 53.95, indicating a robust response of spinach to the applied ultrasound energy. The relative increase rate *b* parameter was moderately significant at 165.29 J/L, while the initial ECR value *y_0_* was close to maximum, showing that spinach reached a high level of conductivity quickly. Lollo Rosso demonstrated a similar but slightly lower response, with the *a* parameter at 0.36 and *E_0_* at 118.70 J/L, suggesting a lower energy requirement to reach the inflection point compared to spinach. The *b* parameter was significantly lower at 40.32 J/L, indicating a slower increase rate near the inflection point, and *y_0_* was lower than that of spinach, reflecting a less pronounced initial response. Chopped loose leaf lettuce showed significant variability, particularly in parameters *b* and *E_0_*. The *b* value was extremely high with a correspondingly large standard error, which suggests the model's fit for this type of lettuce was less reliable, likely due to the uneven distribution of energy absorption across the sample. The *a* parameter was lower at 0.16, and *y_0_* was also relatively modest, indicating a less responsive material under ultrasound treatment. Chopped romaine lettuce exhibited a higher *a* value of 0.53, suggesting a strong response to ultrasound energy. However, similarly to chopped loose leaf lettuce, the *b* and *E_0_*​ parameters had higher variability. This means the response for romaine lettuce is less predictable, likely due to the structure of lettuce causing uneven energy distribution.

**Table 1:** Estimated parameters of the 4-parameter sigmoidal Gompertz model for electro-conductivity rate of selected leaf greens after ultrasound treatment.

Produce	Parameters	Estimate	Std. Error	t	P
Spinach	*a*	0.39	0.06	6.79	<0.0001
	*b*	165.29	73.31	2.25	0.0407
	*E_0_*	298.34	53.95	5.53	<0.0001
	*y_0_*	0.35	73.31	9.79	<0.0001
Lollo Rosso	*a*	0.36	0.05	7.23	<0.0001
	*b*	40.32	17.86	2.26	0.0405
	*E_0_*	118.70	21.64	5.48	<0.0001
	*y_0_*	0.15	0.04	3.47	0.0038
Chopped loose leaf lettuce	*a*	0.16	0.02	5.51	<0.0001
	*b*	8.14	4.49 × 10^8^	1.81 × 10^-8^	1.0000
	*E_0_*	154.63	4.07 × 10^8^	3.80 × 10^-7^	1.0000
	*y_0_*	0.25	0.02	10.95	<0.0001
Chopped romaine lettuce	*a*	0.53	0.18	3.06	0.0085
	*b*	244.47	175.83	1.39	0.1861
	*E_0_*	333.51	141.98	2.35	0.0340
	*y_0_*	0.25	0.11	2.18	0.0465

* *Y(E)* represents ECR (%) at input energy density *E*; *E_0_* represent input energy density at the inflection point; *Y_0_* represents the asymptotic amount of ECR that occurs as E decreases indefinitely; *a* represents the asymptotic amount of ECR increase that occurs as *E* increases indefinitely, and *1/b* represent the relative increase rate at *E_0_* .

These results indicate that different types of leafy greens respond differently to ultrasound treatment, with spinach and Lollo Rosso showing more consistent and predictable increases in ECR, while chopped loose leaf and romaine lettuces demonstrate more complex and less uniform responses. This variability might be attributed to differences in leaf structure, water content, and overall tissue composition, which affect how energy is absorbed and distributed during the treatment.

### 3.2 The effect of ultrasound treatment on quality of fresh-cut iceberg lettuce and romaine lettuce during cold storage

#### 3.2.1 Texture changes during cold storage

Since the properties of iceberg lettuce or romaine lettuce vary across the leaf, the fresh-cut lettuce slices were grouped into rib and leaf portions for quality analysis. The first study was carried out to investigate the effect of ultrasound on the texture of fresh-cut iceberg and romaine lettuce. The maximum force of the mid rib part was significantly greater than that of leaf blade (P < 0.05), for both Romaine lettuce and iceberg lettuce (Table 2).

**Table 2:** Texture analysis of lettuce treated or not by ultrasound and stored at 1 °C.

Produce	Storage time (Day)	Maximum force (N)			
		Rib part		Leaf part	
		Non-Ultrasound	Ultrasound	Non-Ultrasound	Ultrasound
Iceberg lettuce	0	112.70^aA^	104.54^aA^	35.22^aA^	27.41^aA^
	7	154.74^aA^	124.33^aA^	58.50^bA^	59.44^bA^
	14	96.97^aA^	112.27^aA^	30.21^aA^	33.87^aA^
	21	100.06^aA^	75.97^aA^	44.97^aA^	42.81^aA^
Romaine lettuce	0	153.32^aA^	159.71^aA^	32.75^aA^	52.86^bB^
	7	181.73^aA^	146.83^aA^	24.86^aA^	36.89^abA^
	14	170.31^aA^	151.45^aA^	32.80^aA^	25.56^aA^
	21	163.37^aA^	163.80^aA^	38.25^aA^	38.94^abA^

*Lowercase letters represent comparisons among different storage times for a single treatment; uppercase letters represent comparisons among treatments for a single storage time (P < 0.05).

There were no significant changes (P > 0.05) in maximum force of the leaf blade and mid rib parts after storage for 21 days for both varieties of lettuce, and there were no significant differences (P > 0.05) between ultrasound treatment and water only wash during storage. In agreement with Yu et al. [21], we also found that ultrasound-treated samples did not exhibit deterioration during storage, and under certain conditions, it delayed enzymatic browning and maintained better overall quality.

#### 3.2.2 Loss of tissue integrity during cold storage

We investigated the effect of ultrasonication on the electrolyzed leakage of fresh-cut iceberg and romaine lettuce to assess tissue integrity after 21 days of storage (Table 3). There were no significant differences (P > 0.05) in the electrolyte leakage of iceberg lettuce and romaine lettuce between ultrasound treatment and water only wash. However, the ECR of fresh-cut romaine lettuce from the ultrasound treatment was lower than water alone wash at day 14. This difference might be caused by ultrasonic growth stimulation on the plant. Ananthakrishnan et al. [22] also reported that ultrasonic treatment (0.5–2 min) stimulated multiple shoot regeneration to high levels in vitro from recalcitrant cotyledon explants of commercial squash (Cucurbita pepo L.) cultivars. In that study, longer periods of ultrasound (5–10 min) caused further surface erosion.

**Table 3:** Tissue leakage analysis of lettuce treated or not by ultrasound and stored at 1 °C.

Storage time (Day)	Tissue leakage electro-conductivity rate (%)			
	Iceberg lettuce		Romaine lettuce	
	Non-Ultrasound	Ultrasound	Non-Ultrasound	Ultrasound
0	0.49^bA^	0.37^bB^	0.44^abA^	0.50^aA^
7	0.20^aA^	0.22^aA^	0.25^aA^	0.27^aA^
14	0.45^bA^	0.38^bA^	0.60^bA^	0.36^aB^
21	0.52^bA^	0.56^cA^	0.93^cA^	0.79^bA^

*Lowercase letters represent comparisons among different storage times for a single treatment; uppercase letters represent comparisons among treatments for a single storage time (P < 0.05).

ECR for romaine and iceberg lettuce significantly decreased within 7 days (P < 0.05), and then increased afterwards. The decrease in tissue electrolyte leakage during the first 7 days may be attributed to the self-recovery mechanism of damaged membrane during the early stages of cold storage. This self-recovery process has been described in a previous study on the postharvest biology and quality of fresh-cut cilantro leaves under controlled atmospheric packaging [23]. In the latter stages during storage after 7 days, the increase in electrolyte leakage probably accounts for irreversible membrane damage and deletion of O_2_ from respiration [24].

Electrolyte leakage is closely related to cell membrane integrity and permeability [19], and the results of our study indicate that ultrasonication at 81 W/L did not injure lettuce cell and tissue.

#### 3.2.3 Hunter color of fresh-cut iceberg lettuce and romaine lettuce

Color is a critical quality attribute for fresh-cut produce, as it directly influences consumer perception and acceptance [25]. The L value (lightness), hue angle, and chroma provide objective measures of color changes in food products, reflecting their freshness and visual appeal. In this study, the colors of leaves and ribs of romaine lettuce and iceberg lettuce were measured separately.

For iceberg lettuce, the L values significantly increased (P < 0.05) during storage for all iceberg lettuce samples (Table 3). Studies have shown that such increases can result from cell dehydration, tissue senescence, or oxidation processes that alter the reflective properties of leaf tissues [26]. However, there were no significant differences (P > 0.05) in the L values of leaves and ribs between ultrasound treatment and water only wash for iceberg lettuce after storage for 21 days. These results suggest that ultrasound washing neither enhances nor degrades the lightness compared to conventional washing methods. This finding aligns with prior research indicating that mild ultrasound treatments effectively clean produce surfaces without adversely affecting color parameters [27]. The hue angle values from leaves and ribs of iceberg lettuce treated by ultrasound and water only did not experience a significant change (P > 0.05) during storage (Table 4). There were no significant changes (P > 0.05) in chroma values of leaves and ribs between ultrasound treatment and water r wash only for iceberg lettuce during storage.

**Table 4:** Effect of ultrasound on the Hunter color of fresh-cut iceberg lettuce and romaine lettuce.

Produce	Part	Treatment	Storage time (Day)	L	a	b	Hue	Chroma
Iceberg	Leaf	Non-Ultrasound	0	69.25^aA^	−12.37^cA^	29.15^aA^	−1.17^aA^	31.69^abA^
			7	77.23^bA^	−9.14^cA^	20.84^aA^	−1.16^aA^	22.77^aA^
			14	94.96^cA^	−29.76^aA^	39.93^bA^	−0.93^dA^	49.80^cA^
			21	96.70^cA^	−18.42^bA^	30.53^abA^	−1.03^cA^	35.67^bA^
		Ultrasound	0	71.01^aA^	−6.92^bB^	23.12^abA^	−1.29^aB^	24.18^abA^
			7	76.08^bA^	−8.60^bA^	20.38^aA^	−1.18^bA^	22.14^aA^
			14	90.21^cA^	−17.55^aB^	31.76^bB^	−1.07^cB^	36.36^cB^
			21	100.71^cA^	−17.91^aA^	28.60^abA^	−1.01^dA^	33.75^bcA^
	Rib	Non-Ultrasound	0	71.07^aA^	−0.81^aA^	8.85^bA^	−0.85^aA^	8.95^bA^
			7	71.76^aA^	−2.80^aA^	11.43^bA^	−1.34^aA^	11.80^bA^
			14	104.92^bA^	−3.25^aA^	0.69^aA^	−0.45^aA^	5.27^aA^
			21	100.29^bA^	−0.90^aA^	3.82^aA^	-0.1^5aA^	4.14^aA^
		Ultrasound	0	72.93^aA^	−1.93^aA^	8.40^bA^	−1.35^aA^	8.63^bcA^
			7	72.72^aA^	−1.44^aA^	9.71^cA^	−0.80^aA^	9.85^cA^
			14	97.21^bB^	−2.50^aA^	4.26^ab^	−0.48^aA^	5.57^abA^
			21	97.25^bA^	−0.56^aA^	3.37^aA^	−0.78^aA^	3.45^aA^
Romaine	Leaf	Non-Ultrasound	0	80.23^cA^	−21.70^bA^	30.26^abA^	−0.95^abA^	37.27^abA^
			7	50.57^aA^	−19.82^bA^	32.35^abA^	−1.01^aA^	38.03^abA^
			14	65.76^bA^	–22.70^bA^	23.85^aA^	−0.81^cA^	32.94^aA^
			21	82.85^cA^	−26.92^aA^	34.66^bA^	−0.91^bA^	43.93^bA^
		Ultrasound	0	87.59^bA^	–22.25^bA^	35.47^aA^	−1.00^aA^	41.97^aA^
			7	55.50^aA^	−19.53^bA^	35.15^aA^	−1.06^aA^	40.26^aA^
			14	79.50^abB^	−29.97^aB^	38.98^aB^	−0.91^bB^	49.19^aB^
			21	81.90^bA^	−25.70^bA^	32.93^aA^	−0.91^bA^	41.81^aA^
	Rib	Non-Ultrasound	0	98.85^cA^	1.11^bA^	0.39^abA^	0.33^bA^	1.53^aA^
			7	65.32^aA^	−3.59^aA^	12.89^cA^	−1.29^aA^	13.43^cA^
			14	93.92^bA^	−3.21^aA^	3.17^bA^	−0.25^abA^	4.86^bA^
			21	101.88^cA^	4.37^cA^	0.25^aA^	0.33^bA^	4.78^bA^
		Ultrasound	0	102.33^cA^	1.07^bA^	1.82^aA^	0.36^cA^	2.82^aA^
			7	64.00^aA^	−3.71^aA^	13.45^cA^	−1.30^aA^	13.97^cA^
			14	94.09^bA^	−4.28^aA^	5.69^bA^	−0.93^abA^	7.21^bA^
			21	102.29^cA^	5.59^cA^	0.94^aA^	0.12^bcA^	6.33^bA^

*Lowercase letters represent comparisons among different storage times for a single treatment; uppercase letters represent comparisons among treatments for a single storage time (P < 0.05).

In romaine lettuce, the L values significantly decreased after one-week of storage (P < 0.05), and then increased again from day-7 for all romaine lettuce samples. The L values of ribs were significantly greater than leaves for romaine lettuce (P < 0.05). Results were similar for romaine lettuce after 21 days of storage, and no significant differences (P > 0.05) were found in the L values of leaves and ribs between ultrasound treatment and water only wash (Table 4). The hue angle values from leaves and ribs of the romaine lettuce washed by ultrasound and water only did not experience a significant change (P > 0.05) during storage. There were no significant changes (P < 0.05) in chroma values of leaves and ribs between the two treatments for romaine lettuce after 21 days of storage.

For hue angle and chroma, the stability observed during storage for both lettuce types underscores the treatments’ ability to maintain color integrity. This stability might be linked to the absence of significant enzymatic browning or pigment degradation under the controlled storage conditions. Previous research suggests that storage temperatures around 4 °C can slow down enzymatic activities like polyphenol oxidase (PPO) action, which is responsible for browning and color changes in fresh-cut produce [28].

#### 3.2.4 Sensory quality of fresh-cut iceberg lettuce and romaine lettuce during cold storage

The sensory quality of fresh-cut lettuce is a multifaceted attribute, encompassing visual appearance, texture, odor, and overall acceptability. The sensory evaluation was conducted separately for leaves and ribs for romaine lettuce and iceberg lettuce.

**Fig. 4:**
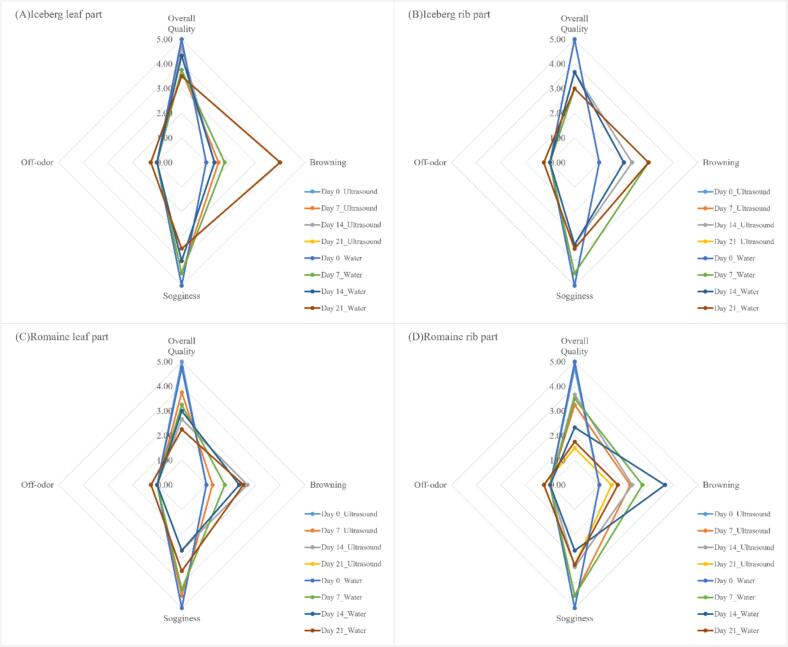
Effect of ultrasonication on the sensory attributes of fresh-cut lettuce treated at 34 kHz and acoustic power density of 81 W/L and stored at 1 °C for 14 days. (A) Iceberg leaf part, (B) Iceberg rib part, (C) Romaine leaf part, and (D) Romaine iceberg rib part.

Results showed no significant differences (P > 0.05) in overall quality, browning index, sogginess index and off-odor index between two wash methods for both varieties of lettuce after storage for 21-day storage (Fig. 4). This demonstrates that ultrasound treatment does not introduce perceivable differences in quality parameters compared to water-only washing. This finding supports the notion that ultrasound washing is a non-invasive method that effectively sanitizes fresh-cut produce while preserving its sensory properties [29]. In addition, the score of overall quality of all samples was over 3 after storage. This result reflects the efficiency of cold storage in preserving sensory attributes and mitigating spoilage effects.

### 3.3 Effect of ultrasound on the quality of Lollo Rosso (Red leaf lettuce) during cold storage

**Fig. 5:**
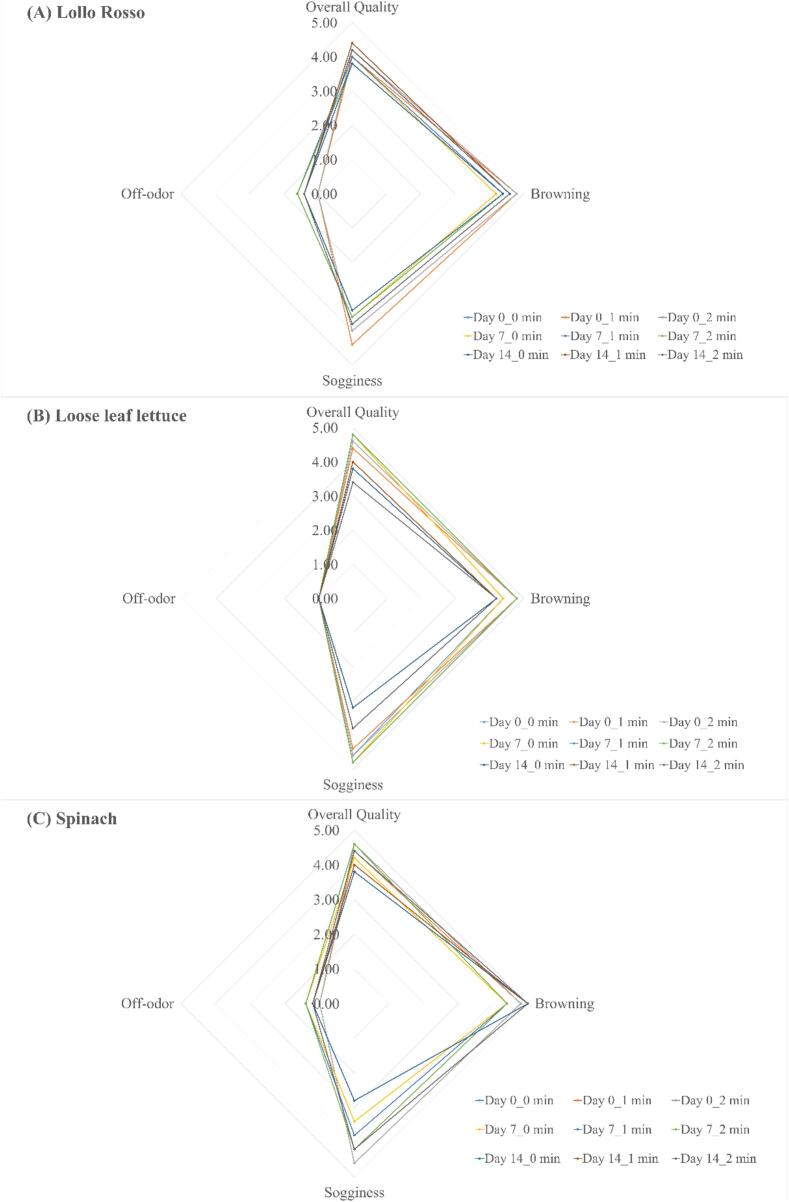
Effect of ultrasonication on the sensory attributes of selected leafy greens at 34 kHz and acoustic power density of 81 W/L during storage at 1 °C. (A) Lollo Rosso lettuce, (B) Loose leaf lettuce, and (C) Spinach.

Fig. 5 shows no significant differences (P > 0.05) in overall quality, discoloration index, sogginess index, and off-odor index between the two wash methods after 14 days of storage. The overall quality of the samples treated by ultrasound for 2 min did not significantly change (P > 0.05) after storage, and the overall quality score of all samples was over 3.

**Fig. 6:**
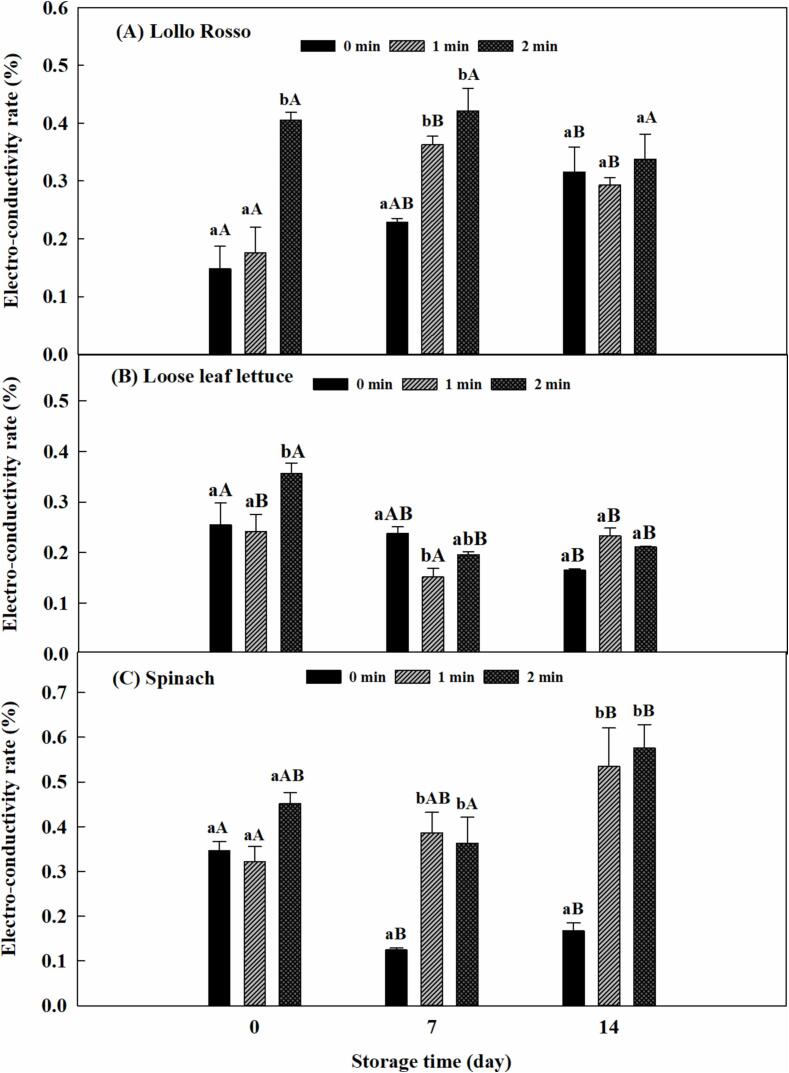
Effect of ultrasonication on sensory attributes of selected leafy greens at 34 kHz and acoustic power density of 81 W/L during storage at 1 °C. (A) Lollo Rosso, (B) Loose leaf lettuce, and (C) Spinach. Lowercase letters represent comparisons among treatment time within the same storage time; uppercase letters represent comparisons among different storage times within the same treatment time (P < 0.05).

Lollo Rosso samples treated by ultrasound for 2 min had a significantly higher ECR than water only wash (P < 0.05), while there were no significant differences (P > 0.05) in the ECR of Lollo Rosso treated by both ultrasound for 1 min and water only at days 0 and 14 (Fig. 6A). The ECR for water ultrasonication for 1 min significantly increased from day 0 to day 7 during storage (P < 0.05), while the ECR for ultrasonication for 1 min did not change for Day 14 days compared to Day 7.

Unlike iceberg lettuce, romaine lettuce, and spinach, the ECR of Lollo Rosso continuously increased over 14 days of storage even for the sample treated with water only. This may indicate the self-repair system is weak or absent in this produce. In addition, the Lollo Rosso sample treated by 1-min ultrasound showed a higher value of ECR on day 7 than on day 0, which may also indicate that ultrasonication did not stimulate the self-repair process of Lollo Rosso tissue.

### 3.4 Effect of ultrasound on the quality of loose-leaf lettuce during cold storage

The effect of ultrasound on the quality of loose-leaf lettuce (Fig. 5B) showed no significant differences (P > 0.05) in the overall quality, discoloration index, sogginess index, and off-odor index ultrasound wash and water only wash during 14 days of storage. Overall quality of the samples did not change significantly after 14 days of storage (P > 0.05). Quality scores of all samples were over 3.

Fig. 6B shows no significant differences (P > 0.05) in ECR of loose-leaf lettuce between ultrasonication for 2 min and water only at day 7 and day 14, while loose leaf lettuce samples treated by ultrasound for 2 min had significantly higher ECR (P < 0.05) than water only wash on day 0, which means that a 2-min ultrasonication may injure cells and tissue, and lead to tissue fluid leakage. The ECR of ultrasonicated samples decreased significantly (P < 0.05) after 7 days. The difference in ECR changes between ultrasound-treated samples and water-washed samples may account for the difference in the activation of the self-repair system in loose leaf lettuce. The ECR of water washed samples did not change after 7 days, and then decreased significantly (P < 0.05) after 14 days of storage.

### 3.5 Effect of ultrasound on the quality of spinach during cold storage

We evaluated the effect of ultrasonication on the quality of baby spinach leaves (Fig. 5C), and results showed no significant differences (P > 0.05) in overall quality, discoloration index, sogginess index and off-odor index between ultrasound treatment and water only wash after

storage for 14 days. The samples washed with water only had a lower sogginess index after 14 days. In addition, overall quality scores of all samples were over 3 after 14 days.

There were no significant differences (P > 0.05) in ECR between ultrasound treatment and water only wash at day 0. However, after 7 days of storage, the samples treated by ultrasound had higher ECR than water only wash (Fig. 6C). This shows that ultrasonication might inhibit the self-repair process of spinach leaf tissue.

It should be noted that the APD level of 81 W/L determined in this work represents the upper ultrasonic power level that retains acceptable produce quality while enhancing the combined ultrasound and sanitizer treatment for produce sanitation. The main purpose of applying ultrasound in fresh produce sanitation is to utilize cavitation-triggered events, such as water jets, strong local macro-streaming, and microstreaming, to dislodge bacterial cells attached to produce surfaces with microscale roughness (which prevents sanitizers from reaching the cells harbored in such areas). Once the attached bacterial cells are removed from produce surfaces, they can be easily killed by a sanitizer, even at low concentrations. Therefore, enhanced sanitation efficacy is achieved through the synergy between sonication and the sanitizer. In a previous study conducted by our group, we performed combined ultrasound and chlorine sanitation tests with an acoustic power density (APD) of 79.41 W/L. Indeed, the introduction of ultrasound resulted in an additional 68 % inactivation of inoculated E. coli cells on produce surfaces compared to treatment with chlorine alone [30].

## 4 Conclusions

The overall quality scores of baby spinach, Lollo Rosso, loose leaf lettuce, iceberg lettuce and romaine lettuce remained unchanged when treated with ultrasound at an acoustic power density (APD) of less than 80 W/L for less than 2 min, while the electro-conductivity rate (ECR) of all four salad leaves increased over treatment time. The quality changes of the five selected leafy produce after ultrasound treatment and during storage were comparable to water only wash and all were above acceptable levels. Future research may explore the long-term effects of ultrasound on produce quality and shelf life, optimize treatment parameters for maximum efficacy and study a wider range of produce commodities.

## CRediT authorship contribution statement

**Bin Zhou:** Writing – original draft, Investigation, Formal analysis, Data curation, Conceptualization. **J. Atilio de Frias:** Writing – review & editing, Investigation, Formal analysis, Data curation. **Yaguang Luo:** Writing – review & editing, Methodology, Formal analysis, Data curation. **Jorge M. Fonseca:** Writing – review & editing, Methodology, Formal analysis, Data curation. **Hao Feng:** Writing – review & editing, Supervision, Funding acquisition, Conceptualization.

## Declaration of competing interest

The authors declare that they have no known competing financial interests or personal relationships that could have appeared to influence the work reported in this paper.

## References

[1] de Chiara M.L.V., Castagnini J.M., Capozzi V. (2024). Cutting-edge physical techniques in postharvest for fruits and vegetables: unveiling their power, inclusion in ‘hurdle’ approach, and latest applications. Trends Food Sci. Technol..

[2] Zhou B., Feng H., Luo Y. (2009). Ultrasound enhanced sanitizer efficacy in reduction of Escherichia coli O157:H7 population on spinach leaves. J. Food Sci..

[3] H. Lee, B. Zhou, H. Feng, Power ultrasound treatment of fruits and fruit products, In: A. Rosenthal, R. Deliza, J. Welti-Chanes, G. Barbosa-Cánovas (eds), Fruit Preservation, Food Engineering Series, Springer, New York, NY, 2020, pp.311-333, https://doi.org/10.1007/978-1-4939-3311-2_11.

[4] Ma T., Wang J., Wang L., Yang Y., Yang W., Wang H., Lan T., Zhang Q., Sun X. (2020). Ultrasound-combined sterilization technology: an effective sterilization technique ensuring the microbial safety of grape juice and significantly improving its quality. Foods.

[5] Feng Y., Suo K., Zhang Y., Yang Z., Zhou C., Shi L., Chen W., Wang J., Wang C., Zheng Y. (2024). Ultrasound synergistic slightly acidic electrolyzed water treatment of grapes: impacts on microbial loads, wettability, and postharvest storage quality. Ultrason. Sonochem..

[6] Flint E.B., Suslick K.S. (1991). The temperature of cavitation. Science.

[7] Herbert E., Balibar S., Caupin F. (2006). Cavitation pressure in water. Phys. Rev. E.

[8] Suslick K.S., Flannigan D.J. (2008). Inside a collapsing bubble: sonoluminescence and the conditions during cavitation. Annu. Rev. Phys. Chem..

[9] Miller D.L. (1979). Cell death thresholds in Elodea for 0.45–10 MHz ultrasound compared to gas-body resonance theory. Ultrasound Med. Biol..

[10] Miller M.W., Miller D.L., Brayman A.A. (1996). A review of in vitro bioeffects of inertial ultrasonic from a mechanistic perspective. Ultrasound Med. Biol..

[11] Martin C.J., Gemmell H.G. (1979). A study of ultrasonically induced pulsations of gas-filled channels in Elodea. Phys. Med. Biol..

[12] Zhou B., Lee H., Demirci A., Ngadi M. (2012). Food Decontamination Novel Methods and Applications.

[13] Prokic M., Feng H., Barbosa-Cánovas G.V., Weiss J. (2010). Ultrasound Technologies for Food and Bioprocessing.

[14] J. Yu, Ultrasonication as an abiotic elicitor-effects on antioxidant capacity and overall quality of romaine lettuce (Master’s thesis, University of Illinois at Urbana-Champaign), 2014, Permalink: http://hdl.handle.net/2142/49484.

[15] Fan K., Wu J., Chen L. (2021). Ultrasound and its combined application in the improvement of microbial and physicochemical quality of fruits and vegetables: a review. Ultrason. Sonochem..

[16] Fan X., Sokorai K.J. (2005). Assessment of radiation sensitivity of fresh-cut vegetables using electrolyte leakage measurement. Postharvest Biol. Technol..

[17] Lester G.E., Saftner R.A. (2008). Marketable quality and phytonutrient concentrations of a novel hybrid muskmelon intended for the fresh-cut industry and its parental lines: Whole-fruit comparisons at harvest and following long-term storage at 1 or 5° C. Postharvest Biol. Technol..

[18] Azodanlou R., Darbellay C., Luisier J.L., Villettaz J.C., Amadò R. (2003). Development of a model for quality assessment of tomatoes and apricots. LWT- Food Sci. Technol..

[19] Marangoni A.G., Palma T., Stanley D.W. (1996). Membrane effects in postharvest physiology. Postharvest Biol. Technol..

[20] Umaña M., Calahorro M., Eim V., Rosselló C., Simal S. (2022). Measurement of microstructural changes promoted by ultrasound application on plant materials with different porosity. Ultrason. Sonochem..

[21] Yu J., Engeseth N.J., Feng H. (2016). High intensity ultrasound as an abiotic elicitor—effects on antioxidant capacity and overall quality of romaine lettuce. Food Bioproc. Tech..

[22] Ananthakrishnan G., Xia X., Amutha S., Singer S., Muruganantham M., Yablonsky S., Fischer E., Gaba V. (2007). Ultrasonic treatment stimulates multiple shoot regeneration and explant enlargement in recalcitrant squash cotyledon explants in vitro. Plant Cell Rep..

[23] Luo Y., Wachtel M.R., McEvoy J.L., Kim J., Hung Y. (2004). Package atmosphere affects postharvest biology and quality of fresh-cut cilantro leaves. HortSci..

[24] Wang H., Feng H., Luo Y. (2004). Microbial reduction and storage quality of fresh-cut cilantro washed with acidic electrolyzed water and aqueous ozone. Food Res. Int..

[25] Clydesdale F.M., Ahmed E.M. (1978). Colorimetry-methodology and applications. Crit. Rev. Food Sci. Nutr..

[26] Barrett D.M., Beaulieu J.C., Shewfelt R. (2010). Color, flavor, texture, and nutritional quality of fresh-cut fruits and vegetables: desirable levels, instrumental and sensory measurement, and the effects of processing. Crit. Rev. Food Sci. Nutr..

[27] Chemat F., Rombaut N., Meullemiestre A., Turk M., Perino S., Fabiano-Tixier A.S., Abert-Vian M. (2017). Review of green food processing techniques. Innov. Food Sci. Emerg. Technol..

[28] Oms-Oliu G., Soliva-Fortuny R., Martín-Belloso O. (2008). Using polysaccharide-based edible coatings to enhance quality and antioxidant properties of fresh-cut melons. LWT-Food Sci. Technol..

[29] Guo Y., Wu B., Guo X., Liu D., Wu P., Ma H., Pan Z. (2021). Ultrasonication and thermosonication blanching treatments of carrot at varying frequencies: effects on peroxidase inactivation mechanisms and quality characterization evaluation. Food Chem..

[30] Zhou B., Feng H., Pearlstein A.J. (2012). Continuous-flow ultrasonic washing system for fresh produce surface decontamination. Innov. Food Sci. Emerg. Technol..

