# Supplementary material for: Impact of power ultrasound on the quality of leafy green produce through a multifrequency, multimode, modulated system
Source: Ultrason Sonochem. 2025 Jan 2;113:107221. doi: 10.1016/j.ultsonch.2024.107221 (PMC11758820; doi:10.1016/j.ultsonch.2024.107221)
Supplement: Supplementary Data 1 [file mmc1.docx]

**Supplementary Fig S1.** Temperature change of washing solution during ultrasound treatment.
